# Acupuncture promotes nerve repair through the benign regulation of mTOR‐mediated neuronal autophagy in traumatic brain injury rats

**DOI:** 10.1111/cns.14018

**Published:** 2022-11-24

**Authors:** Sisi Zhao, Shiqi Wang, Luxi Cao, Hai Zeng, Shujun Lin, Zhuowen Lin, Minan Chen, Mingmin Zhu, Zhao Pang, Yimin Zhang

**Affiliations:** ^1^ School of Traditional Chinese Medicine Jinan University Guangzhou China; ^2^ Medical College of Acupuncture‐Moxibustion and Rehabilitation Guangzhou University of Chinese Medicine Guangzhou China; ^3^ Medical Administration Division The First Affiliated Hospital of Jinan University Guangzhou China

**Keywords:** acupuncture, mTOR/ULK1, neuronal autophagy, traumatic brain injury (TBI)

## Abstract

**Aims:**

Recent investigations have already proved the neuroprotective efficacy of acupuncture in clinical practice in the treatment of neurological diseases, such as traumatic brain injury (TBI). Since growing evidence has suggested that neuronal autophagy was involved in multiple stages of TBI, this study aims to clarify the autophagy mediating mechanism underlying the neuroprotective effect of acupuncture in TBI rats.

**Methods:**

Three experiments were carried out to detect changes in neuronal autophagy and identify the potential molecular mechanism underlying the neuroprotective effect of acupuncture for TBI treatment. Feeney's free‐falling epidural impingement method was used to establish the moderate TBI rat model; modified neurological severity scoring (mNSS) was used for neurological recovery evaluation. Nissl and HE staining were used to examine the histopathological changes. Immunofluorescence was used to detect the LC3‐positive cell rate. The transmission electron microscope (TEM) was used to investigate the morphology and quantity of autophagosomes. Western blotting was used to determine the protein expressions of LC3, p62, beclin1, mTOR, ULK1, p‐mTOR, and p‐ULK1. Quantitative real‐time polymerase chain reaction (qRT‐PCR) was used for gene expressions analysis of LC3 mRNA and p62 mRNA. Co‐immunoprecipitation (CO‐IP) method was used to identify the protein interaction of mTOR and ULK1.

**Results:**

On Day 3 after TBI, acupuncture accelerated the removal of damaged cellular structures by promoting neuronal autophagy; on Day 7 and Day 14 after TBI, acupuncture inhibited neuronal autophagy, preventing excessive autophagy and thus alleviated nerve damage. In addition, the simultaneous treatment with 3‐MA or rapamycin at different stages after TBI attenuated the effect of acupuncture.

**Conclusion:**

Acupuncture has a benign regulatory effect on neuronal autophagy in different stages of TBI, possibly through the mTOR/ULK1 pathway.

## Graphical abstract

Neuronal damage of traumatic brain injury (TBI) rats commonly leads to severe neurological dysfunctions. Pathological results show that cortical neurons in TBI rats exhibit a wide range of degenerative changes, including atrophy, deformation, and disordered alignment, which can be improved by acupuncture. These results raise the question of the underlying molecular mechanism of acupuncture‐induced morphological restoration of neurons in TBI rats.

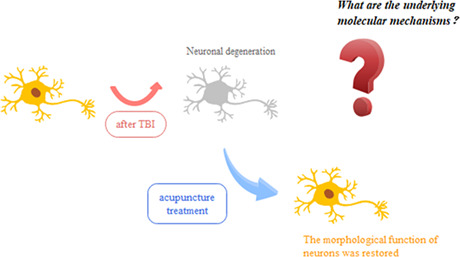

## 1 INTRODUCTION

Traumatic brain injury (TBI)^1^, ^2^ is a mechanical injury with different degrees mainly caused by external physical impact, leading to temporary or even permanent brain function damage. Worldwide, approximately 50 million people have suffered from TBI each year, posing a great threat to global public health.^3^ In particular, China has the greatest number of TBI patients across the world. The pathogenesis of TBI is rather complex, including two stages of primary and secondary injury. It is easy to develop into the second stage if not provided prompt and effective treatment, which causes a poor prognosis and low quality of life (QoL), bringing a huge economic burden to family and society.^4^ Many studies^5^, ^6^, ^7^ have proved that acupuncture was beneficial to TBI in brain function restoration and QoL improvement.

Autophagy is a highly conserved biodegradation pathway, playing an essential role in maintaining cellular homeostasis as well as regulating cell development and survival.^8^ In mammalian cells, three types of autophagy were well established: macroautophagy, microautophagy, and chaperone‐mediated autophagy. Macroautophagy (referred to as autophagy) involves the formation of a double‐membrane vesicle, known as autophagosome, which fuses with lysosomes to form autophagolysosomes^.^ ^9^ Furthermore, the autophagy flux after TBI is time‐dependent^.^ ^10^ At the early phase of TBI, the death of neurons in the ipsilateral cerebral cortex results from the abnormal aggregation of autophagosomes, including the accumulation of autophagy substrate p62 and impaired autophagic flux, caused by lysosome dysfunction.^11^ Rapamycin (mTOR) kinase is a significant target for the sensation and regulation of cell growth and autophagy, regulating signals such as growth factor receptor, hypoxia, ATP, and downstream insulin. The UN51‐like kinase 1(ULK1), a critical factor in the starvation‐induced autophagy pathway, is a protein kinase that strictly regulates the initiation of autophagy.^12^ The mTOR signaling may regulate the initiation, process, and cessation of autophagy by controlling ULK1.^13^ Meanwhile, rapamycin is a known and efficacious mTOR inhibitor.^11^

Recently, researchers confirmed^14^, ^15^ that acupuncture could induce or inhibit autophagy by improving endoplasmic reticulum stress (ERS) and regulating specific autophagy pathways, thus preserving nerve function and ameliorating neurological diseases. However, the specific mechanism remains unclear in acupuncture regulating autophagy to provide a neuroprotective effect after TBI. Therefore, it is vital to explore the benign mechanism of acupuncture on brain neuronal autophagy in TBI rats.

## 2 MATERIALS AND METHODS

### 2.1 Experimental animals

The male Sprague–Dawley rats, weighed 250 ± 20 g and 8 weeks old, were purchased from Weitonglihua Experimental Animal Technology Co., Ltd. All animal experiments were operated in the Experimental Animal Management Center of Jinan University (License Number: SYXK [Guangdong] 2017‐0174). The experiment was conducted after 10 days of adaptive feeding. (Approval No. IACUC‐20200521‐04).

### 2.2 Animal Model

Feeney's freefall epidural impact method was used to establish animal models.^16^ After intraperitoneal injection of 2% sodium pentobarbital solution (0.2 ml/100 g; Sigma), the scalp was cut along the midline of the skull. And a regular circular skull window (with a diameter of 5 mm) was drilled at 2 mm on the right side of the sagittal suture and 1 mm behind the coronal suture. And then, we used a free‐fall impact platform (Zhenghua Biological Instruments Co. Ltd.) with a 20 g weight free‐fall from a height of 30 cm to hit the rivet (4 mm in diameter and 5 mm in length), moderate brain injury resulting in localized right parietal cortex in rats. At last, hemostasis and sutures were performed under aseptic conditions and regular feeding after resuscitation at 37°C.

### 2.3 Acupuncture treatment

Treatment was performed within 24 h after modeling. Selected acupoints: Baihui (GV20), Shuigou (GV26), Fengfu (GV16) through Yamen (GV15) and bilateral Hegu (LI4). The disposable acupuncture needle (0.18 × 15 mm, Shenlong Medical Instrument Co., Ltd.) was inserted 2 mm into the acupoint, and even reinforcing–reducing method was adopted. The needle was retained for 15 min, and each needle was twirled once every 5 min, once a day. The course of treatment was divided into 3 days, 7 days, and 14 days. The model and normal groups both received no treatment, while the model group received fixation as same as the treatment group. All these treatments were performed by the same operator and accord with the principle of random and blindness.

### 2.4 Experimental design

#### 2.4.1 Experiment 1

**FIGURE 1:**
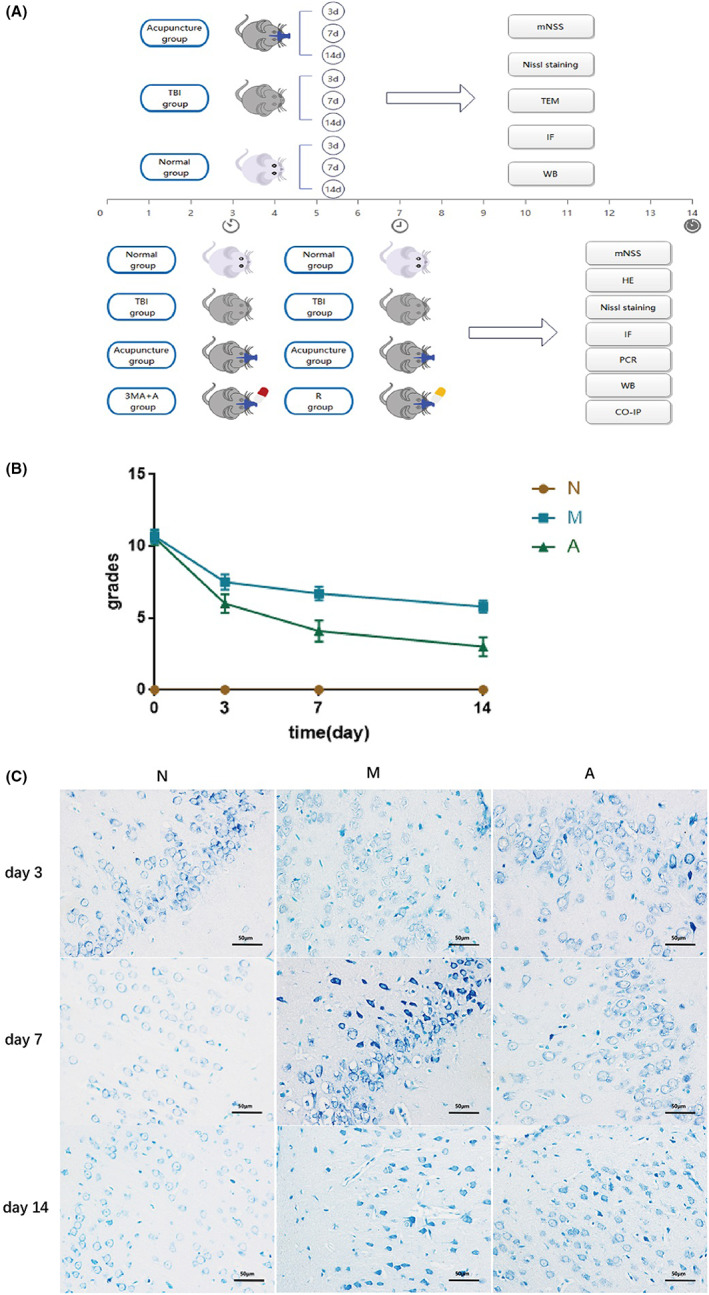
Effects of acupuncture on post‐TBI neural function scores and neuron morphology in cerebral cortex's injured peripheral lesions. (A) Experimental roadmap. (B) The mNSS neural function scores in each group. (C) Nissl staining (bar = 50 μm). All results were presented as mean ± standard deviation. *p < 0.05* defined group difference was statistically significant.

This experiment explored the effect of acupuncture in rat TBI models (Figure 1A). Seventy SPF SD male rats were randomly divided into the normal group (N group, *n* = 10), the TBI model group (M group, *n* = 30), and the TBI+ acupuncture treatment group (A group, *n* = 30). Feeney's free‐fall epidural impingement method was used to establish a moderate TBI rat model. After modeling, the rats in group M and group A were randomly divided into three subgroups by treatment time: 3d (*n* = 10), 7d (*n* = 10) and 14d (*n* = 10). Only group A received acupuncture treatment. The mNSS neurological impairment score was used to evaluate the degree of neurological impairment after modeling but before sampling. Ten rats in each group were used for Nissl staining, transmission electron microscopy, immunofluorescence staining, and Western blot.

#### 2.4.2 Experiment 2

This experiment explored the specific molecular mechanism of acupuncture regulating neuronal autophagy in the cortex at the early stage of TBI (Figure 1A). The early time point of treatment (Day 3) was selected for analysis. Thirty‐two SD rats were randomly divided into the normal group (N group, *n* = 8), the TBI model group (M group, *n* = 8), the TBI+ acupuncture group (A group, *n* = 8), and the TBI+ acupuncture +3MA group (3MA group, *n* = 8). The modeling and treatment methods were the same as in Experiment 1. The 3‐MA group was injected with 3‐methyladenine (3‐MA) (3.5 mg/kg, i.v., AbMole, USA) 30 min after the model establishment. The mNSS neurological impairment scores were used in the same way as in Experiment one. Eight rats in each group were detected by Nissl staining, hematoxylin–eosin staining (HE staining), immunofluorescence staining, Western blot, and co‐immunoprecipitation (CO‐IP).

#### 2.4.3 Experiment 3

This experiment explored the specific molecular mechanism of acupuncture regulating neuronal autophagy in the cortex at the middle stage of TBI (Figure 1A). The intermediate time point of treatment (Day 7) was selected for analysis. Thirty‐two SD rats were randomly divided into the normal group (N group, *n* = 8), the TBI model group (M group, *n* = 8), the TBI+ acupuncture group (A group, *n* = 8), and the TBI+ acupuncture + rapamycin group (R group, *n* = 8). The modeling and treatment method were the same as in experiment one. A 2 mg/kg rapamycin (AbMole, USA) dosage was intraperitoneally injected in the R group rats 30 min after TBI induction. Usage of the mNSS neurological impairment score was the same as in Experiment one. Eight rats in each group were detected by Nissl staining, hematoxylin–eosin staining (HE staining), immunofluorescence staining, qRT‐PCR, Western blot, and co‐immunoprecipitation (CO‐IP).

### 2.5 Neurological function assessment

The modified neurological severity scoring (mNSS) was used for scoring.^17^ The first grading was done immediately after modeling, to select the moderate TBI model rats (7–12 points), and the selected rats were randomized into each group. The second grading was performed on the 3, 7, and 14 days, respectively, to compare the effect of acupuncture on TBI rats at different duration points.

### 2.6 Nissl staining

Nissl staining was used to observe the histopathological changes. The rats were anesthetized by intraperitoneal injection of 2% pentobarbital sodium (0.2 ml /100 g), then infused with 0.9% precooled saline and 4% paraformaldehyde (PFA, pH 7.4). The brain tissues were immersed in 4% paraformaldehyde solution for 24 h before making the wax block, and the wax block was cooled at −20°C after gradient dehydration, cut into 4 μm slices, and stored at room temperature. After dewaxing (xylene i for 20 min, xylene ii for 20 min, anhydrous ethanol i for 5 min, anhydrous ethanol ii for 5 minutes, 75% alcohol for 5 minutes, washed with tap water), slices were stained in toluidine blue staining (toluidine blue dye for 2‐5 min, washed with water, 0.1% acetic acid to slightly differentiate, washed with tap water to stop the reaction), and sealed with neutral gum. Finally, the slices were observed under a microscope, and images were collected.

### 2.7 Hematoxylin–eosin staining (HE staining)

Cortical tissue of TBI rats was sliced into 4 μm; paraffin section staining (dyeing with hematoxylin for 0.5‐1 min, rinsing with tap water, differentiating with 1% hydrochloric acid alcohol, returning 1% ammonia solution to blue for 1 minute, adding eosin solution for final staining, rinsing with running water). After dehydration, neutral gum sealing piece; slices were observed under a microscope, and images were collected.

### 2.8 Immunofluorescence Staining

We used immunofluorescence staining to detect the rate of neuronal LC3‐positive cell. The slices were then dewaxed, repaired by antigen (EDTA antigen repair solution, PH8.0), sealed by hydrogen peroxide (3% hydrogen peroxide solution), and serum. Then, the slices were incubated with primary anti‐Neun (1:500, Servicebio), LC3b (1:1600, Cell Signaling) in an incubation box overnight at 4°C. Next, the HRP‐labeled secondary antibody was dripped at room temperature to cover the tissue for 50 minutes. Then the tissue was tipped with CY3 (Servicebio) and incubated at dark and room temperature, for 10 min. DAPI was used to dye the nuclei, followed by incubation at room temperature for 10 min. Finally, the slices were sealed after spontaneous fluorescence quenching (Servicebio). Slices were observed under a fluorescence microscope (Nikon Eclipse C1), and images were collected.

### 2.9 Transmission electron microscope (TEM)

The morphology and number of autophagosomes in neurons were observed by transmission electron microscopy. Under low temperature, the brain tissue was quickly taken out and placed into an electron microscope fixative solution (Servicebio) for anterior fixation, and 1% osmium for posterior fixation. The tissues are dehydrated step by step in 50%, 70% ethanol, and 80%, 90%, 100% acetone (Acetone National Pharmaceutical Group Chemical Reagent Co., LTD., China). The 100% acetone and epoxy resin Epon812 embedding agent (SPI) were mixed in 1:1 and replaced for 40 min. The Epoxy resin Epon812 embedding agent was soaked at 37°C overnight, and then 60°C polymerization for 48 hours. The resin blocks were made into ultra‐thin slices of 40 ~ 60 nm and dyed successively for 3 min in a saturated uranium acetate (prepared by 70% ethanol and lead dye solution), then rinsed with hydrogen peroxide. The transmission electron microscope (H‐7650 transmission electron microscope, Hitachi) was used for observation, image collection.

### 2.10 qRT‐PCR

The relative expression levels of LC3 mRNA and p62 mRNA in the neurons around the injury lesion in the right cerebral cortex of TBI rats were detected by qRT‐PCR. Total RNA was isolated and extracted. Then, diluted the Total RNA of each sample to 500 ng/mL after concentration determination. And then, 10 μl MIX, 5 μl Total RNA, and 35 μl enzyme‐free water were successively added for reverse transcriptional reaction. The cDNA samples were packed and stored under −20°C or −80°C as needed. Next, 2 μl sample cDNA solution, 8.5 μl sterile water, 1 μl Forward Pimer (10uM), 1 μl Reverse Primer (10 μM), and 12.5 μl TB Green Premix EX Taq ii reagent were added into eight‐well PCR strips respectively. The eight‐well PCR strips were then put into CFX96 real‐time fluorescence quantitative PCR instrument for reaction. And following primers were used: LC3: TCCGAGAAGACCTTCAAACAGC (forward), AAGAAGGCTTGGTTAGCATTGAG (reverse); p62: TTGAGAAGATTCAGAAGGGAGAGTC (forward), TCTTCCTCCTTGGCTTTGTCTC (reverse); β‐actin: TGCTATGTTGCCCTAGACTTCG (forward), GTTGGCATAGAGGTCTTTACGG (reverse). The $2^{-\Delta \Delta C_{t}}$ was used to perform the relative quantification of samples.

### 2.11 Western Blot

Western blot was used to detect protein expressions of LC3, P62, mTOR, ULK1, p‐mTOR, and p‐ULK1 in neurons around the injury lesion in the right cerebral cortex of TBI rats. We add 20ul of protease inhibitor mixture (Beyotime Biotechnology), phosphatase inhibitors (Beyotime Biotechnology), and 0.25 m EDTA (ratio 1:50) to per 1 ml lysate (Servicebio) to prepare cell lysis buffer. The total protein solution was produced from the prepared cell lysate and then stored at −80°C for later use. Protein concentration was measured by the BCA protein quantification test kit (Beyotime Biotechnology). Electrophoresis was performed on polyacrylamide gel (SDS‐PAGE gel kit, Beyotime Biotechnology) and transferred to PVDF membrane (Millipore). 5% skim milk powder solution was used as the sealing solution. After the sealing, the PVDF membrane was placed into an incubation box with the mixture of proportionally diluted primary antibodies (LC3B, SQSTM1/ P62, anti‐rabbit IgG, β ‐actin, mTOR, ULK1, Phospho‐ulk1 (Ser757), Phospho‐mTOR (Ser2448), beclin1 (Cell Signaling Company)), and incubated overnight on a shaker at 4°C. Next, it was incubated in the incubator box with secondary antibody (Cell Signaling) at room temperature for 1 h. ECL luminescent solutions A and B prepared in equal volume were used as developer solutions, ChemiDoc imaging system (BIO‐RAD) was used for protein band imaging and detection, and the images were collected and analyzed. ImageJ was used to detect the gray values of the corresponding protein bands. The ratio between the gray values of the target protein and the gray values of the corresponding internal reference protein was the relative gray values of each target protein.

### 2.12 Co‐Immunoprecipitation (CO‐IP)

Cell lysis solution was extracted from cell lysis buffer (containing protease inhibitor), added with 1 μg antibody, and incubated at 4°C overnight. Then, the above solution was mixed with 10 μl protein A agarose beads and incubated at 4°C for 2–4 h. After the immunoprecipitation reaction, we centrifuged the supernatant at 3000 RPM at 4°C for 3 min and rinsed it with lysis buffer solution 3 times. Finally, the solution was added with 15 μl 2 × SDS loading buffer, boiled for 5 minutes, and then performed Western blot.

### 2.13 Statistical analysis

SPSS 26.0 was applied for data analysis, and all measurements were presented as mean ± standard deviation. One‐way AVOVA was used for comparison between groups. Repeated‐measures ANOVA was used for comparison within groups. Student–Newman–Keuls (SNK) test was used for multiple comparisons for data that exhibit a normal distribution. Tamhane's T2 method was used for data that do not exhibit a normal distribution. *p* < 0.05 was considered to be statistically significant (*α* = 0.05).

## 3 RESULTS

### 3.1 Acupuncture could improve neurological function and neuronal morphology of the right cerebral cortex in TBI rats

First, we assessed neurological deficits of rats after TBI by modified neurological severity score (mNSS). As shown in Figure 1B, the neurological score increased dramatically on the first day after the TBI induction, indicating severe neurological damage and successful TBI modeling. Moreover, the injury degree of TBI rats in each group after modeling was consistent (*p* > 0.05). On the third to fourteenth days, neurological function scores of the model group decreased slightly, while the acupuncture group scores dropped drastically. This result suggested that the TBI rats have limited self‐healing capability, and acupuncture can improve nerve function effectively. In addition, the results of Nissl staining (Figure 1C) showed that neurons in the right cerebral cortex of TBI rats had irregular morphology and atrophy, with fuzzy nuclei and chaotic arrangement, and the staining of Nissl bodies became shallow in the early stages of brain injury (Day 3 after TBI). At the later stage of TBI (Day 7 and Day 14 after TBI), the neurons were damaged excessively, arranged loosely, and their number observably declined. However, under acupuncture intervention, the morphology of neurons remained rather complete. There were less vacuolar degeneration and nuclear pyknosis and more Nissl corpuscles with full shape in neurons. The results above showed that acupuncture treatment could effectively protect cortical neurons on the injured side and relieve neurological damage. Notably, the benefits were more evident after 7 and 14 days of treatment.

### 3.2 Acupuncture improves neurological function by benign regulation of neuronal autophagy

**FIGURE 2:**
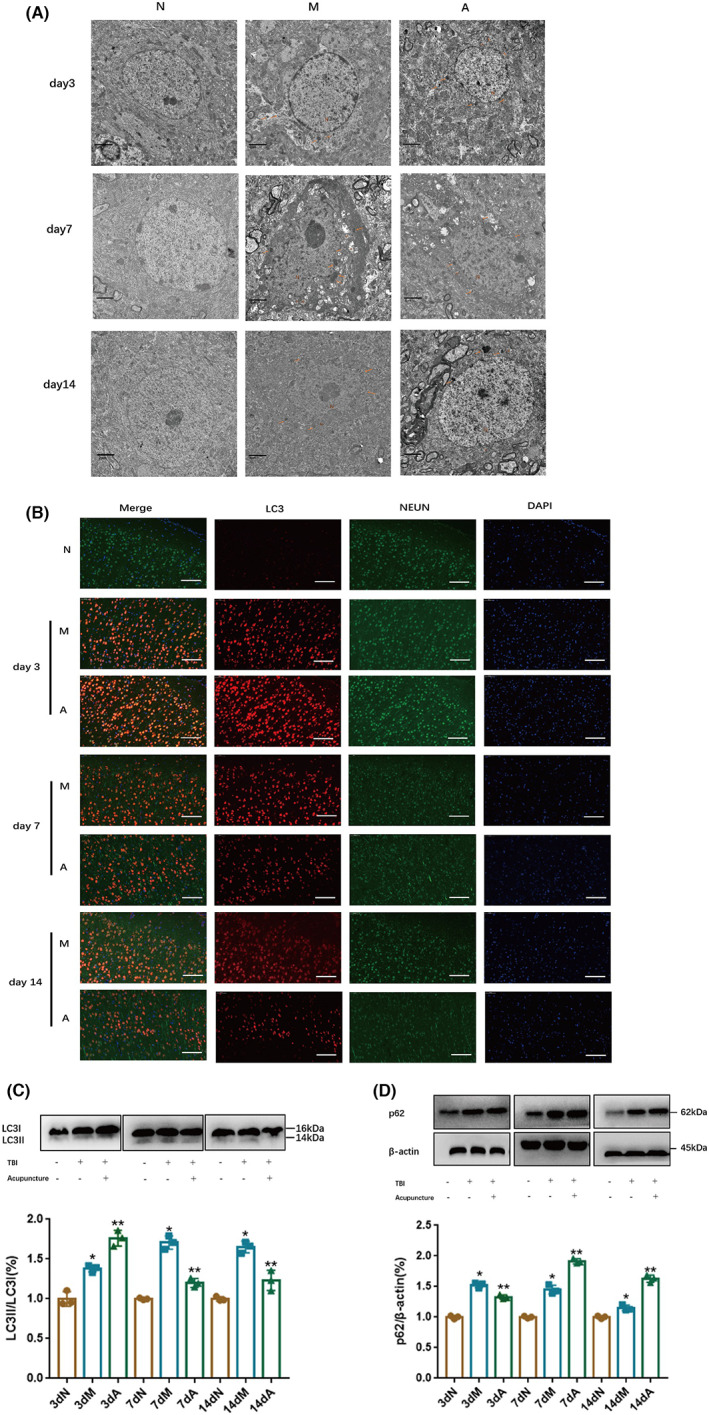
Acupuncture's effect on neuronal autophagy in cerebral cortex's neurons in TBI rats. (A) Each group's ultrastructure of cerebral neurons under the transmission electron microscope (bar = 2 μm); (B) Immunofluorescence results of each group (bar = 50 μm); (C) The experimental image and the bar graph of protein expressions on autophagy marker LC3 (WB); (D) The experimental image and the bar graph of autophagy substrate p62 (WB). All results were presented as mean ± standard deviation. *p* < 0.05 defined group difference was statistically significant; * defined compared with the normal group, and ** defined compared with the model group.

There were morphological and quantitative changes of neuronal autophagosomes on the damaged side of the cerebral cortex in TBI rats under the transmission electron microscope. As shown in Figure 2A, day 3 after TBI, microscopic images exhibited, rough edges in neuron's nuclear, cumulative nuclear heterochromatin with chromatin margination, increased electronic density of cytoplasmic, swollen vacuolar mitochondria, and abundant autophagosomes. At the same time, acupuncture could enhance these changes: both neuronal chromatin and autophagosome increased, and the direction of axon extension was visible in autophagosomes. At the later stage of TBI, the neurons degenerated more evidently. On Day 7 after TBI, chromatin cavitation was observed, with many swollen vacuolated mitochondria and some autophagosomes. On Day 14 after TBI, the nuclear deformation of neurons decreased, as well as the phenomenon of nucleolytic resulting in neuronal degeneration and necrosis appeared. This phenomenon was significantly improved after acupuncture treatment. In detail, the swelling and vacuole of mitochondria improved obviously, the number of autophagosomes reduced, and the boundary of the nuclear and cellular membrane was more distinct. These results suggested that neuronal autophagy may mediate the neuroprotective effect of acupuncture on TBI rats. To confirm this hypothesis, we used the immunofluorescence assay to detect the LC3 positive cell rate of neurons in the injured side of the cerebral cortex, and the Western blot assay to measure the protein expression of autophagy marker LC3 and autophagy substrate p62. As shown in Figure 2B,C, the LC3‐positive cell rate of neurons in the model group increased significantly compared with that of the normal group on the third day after TBI, while acupuncture drastically promoted this change. At the same time, acupuncture downregulated the high level of p62 relative protein expression (Figure 2D) caused by brain injury; and promoted the expression of LC3II/I protein (Figure 2C). On Day 7 and Day 14 after TBI, the LC3‐positive cell rate of neurons slightly lowered in the model group, which was still at a high level (Figure 2B). However, after acupuncture intervention, the LC3‐positive cell rate of neurons decreased obviously, especially on Day 14 after TBI, the neurons were more complete. Furthermore, acupuncture promoted the relative protein expression of p62 and downregulated the expression level of LC3II/I. The above‐mentioned results indicated that the regulation of acupuncture on the cerebral cortex neuronal autophagy was likely to be bidirectional. In the early stage of brain injury (day 3 after TBI), acupuncture removed abnormal organelle and cells by promoting the induction of neuronal autophagy for regular energy supplements. In the middle and late stages of TBI (Day 7 and Day 14 after TBI), acupuncture prevents cell damage by inhibiting neuronal autophagy to keep excessive autophagy.

### 3.3 Acupuncture may regulate neuronal autophagy by modulating mTOR dependent signaling pathway

**FIGURE 3:**
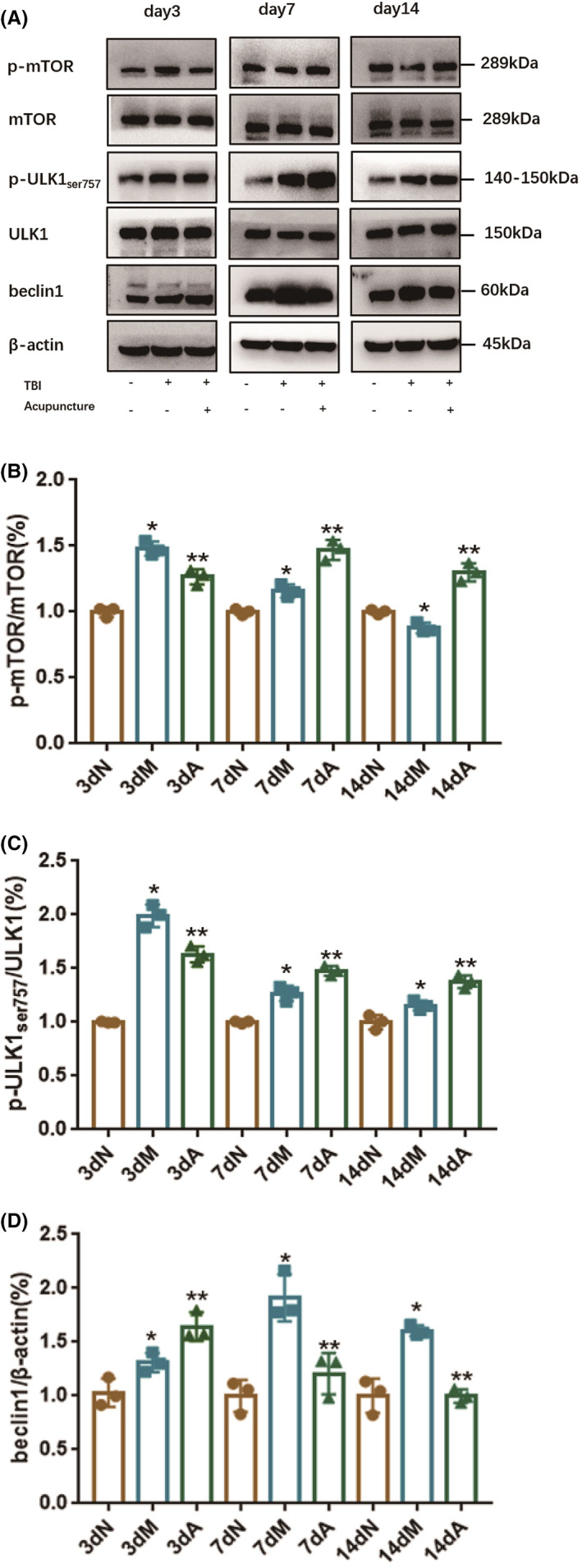
Effect of acupuncture on mTOR pathway in TBI rats. (A) The experimental image of Western blotting; (B) The bar graph of protein expressions on p‐mTOR/mTOR (WB); (C) The bar graph of protein expression on p‐ULK1_ser757_/ULK1 (WB). (D) The bar graph of protein expression on beclin1 (WB). All results were presented as mean ± standard deviation. *p < 0.05* defined group difference was statistically significant; * defined compared with the normal group and ** defined compared with the model group.

Next, the mechanism of acupuncture on benign regulation of neuronal autophagy was discussed. In the injured cortex, the expression levels of p‐mTOR/mTOR (Figure 3A and Figure 3B) and p‐ULK1Ser757 /ULK1 (Figure 3A,C) in TBI rats increased, but decreased significantly after acupuncture treatment on Day 3 after TBI. Acupuncture accelerated the expression of p‐mTOR /mTOR and p‐ULK1ser757/ULK1 on Day 7 and Day 14 in TBI rats. At the same time, the expression of beclin1 (Figure 3A,D) in the cortex was enhanced on the third day after TBI, which was further promoted with acupuncture treatment. On Day 7 and Day 14 after TBI, the therapeutic effect of acupuncture on TBI rats was opposite to that on Day 3. Therefore, we inferred that the benign regulation of acupuncture on autophagy in TBI rats is mediated by the mTOR signaling pathway.

### 3.4 The autophagy inhibitor 3‐MA attenuated the acupuncture efficacy in early TBI rats

**FIGURE 4:**
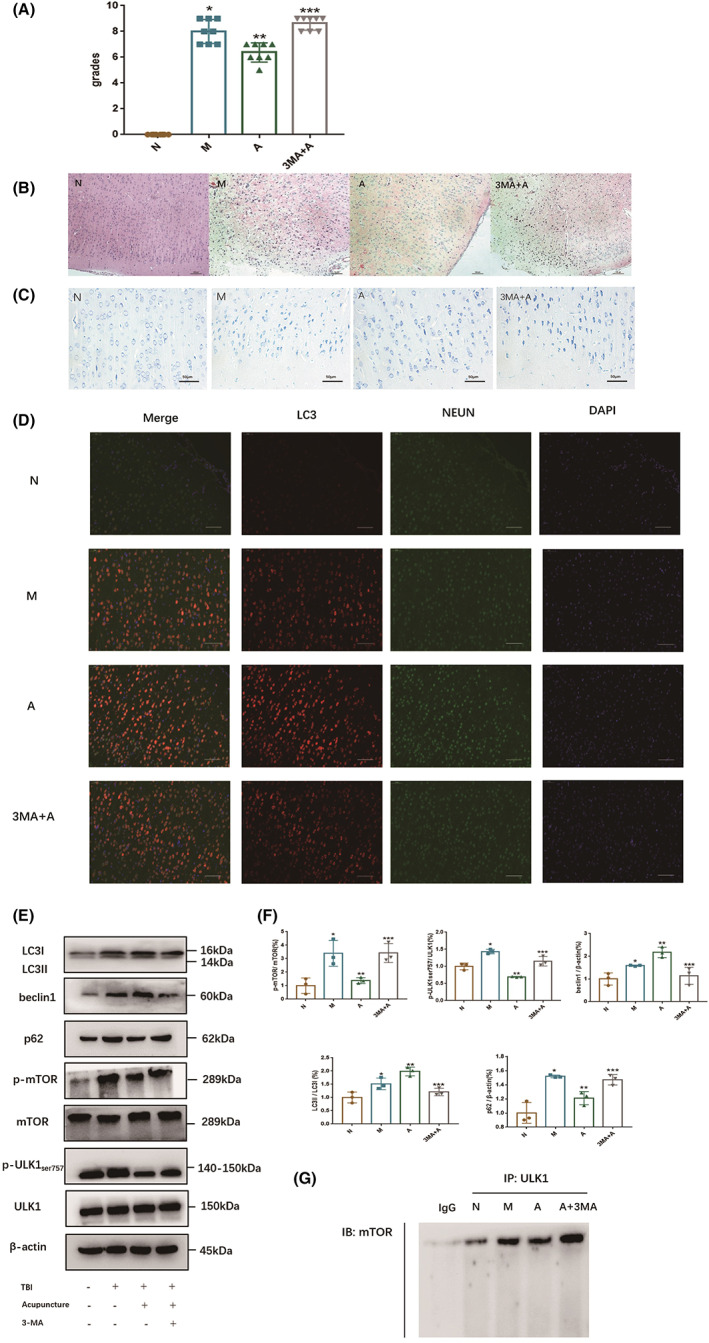
(A) Results of mNSS neurological function scoring; (B) HE staining (bar = 200 μm); (C) Nissl staining image (bar = 50 μm); (D) Immunofluorescence results of each group (bar = 50 μm); (E) (F) The expressions of autophagy‐related proteins were measured with Western blot. (G) The combination of mTOR and ULK1 was determined with co‐immunoprecipitation (CO‐IP). All results were presented as mean ± standard deviation. *p < 0.05* defined group difference was statistically significant; *, **, *** defined compared with the normal group, the model group, and the acupuncture group, respectively.

To investigate the relationship between acupuncture and neuronal autophagy at the early stage of TBI, we detected the indicators related to neuronal autophagy in the cerebral cortex of TBI rats on Day 3 after TBI. Our results showed that 3‐MA weakened the curative effect of acupuncture on TBI rats, with the neurological function (Figure 4A) and morphological recovery of cortical neurons being inhibited (Figure 4B,C). In the meantime, acupuncture treatment increased LC3‐positive cells in neurons of TBI rats (Figure 4D), the multiplication of beclin1, conversion of LC3‐I to LC3‐II, and decreased the expression of autophagy substrate p62, but these effects were counteracted by autophagy inhibitor 3‐MA (Figure 4E,F). Of note, 3‐MA abrogated the acupuncture‐mediated decrease in the p‐mTOR/mTOR ratio and p‐ULK1Ser757/ULK1 ratio. CO‐IP results (Figure 4G) showed that 3‐MA also enhanced the acupuncture‐mediated weakening of the mTOR and ULK1 connection.

### 3.5 The mTOR inhibitor rapamycin can counteract the therapeutic effect of acupuncture on TBI rats by activating autophagy

**FIGURE 5:**
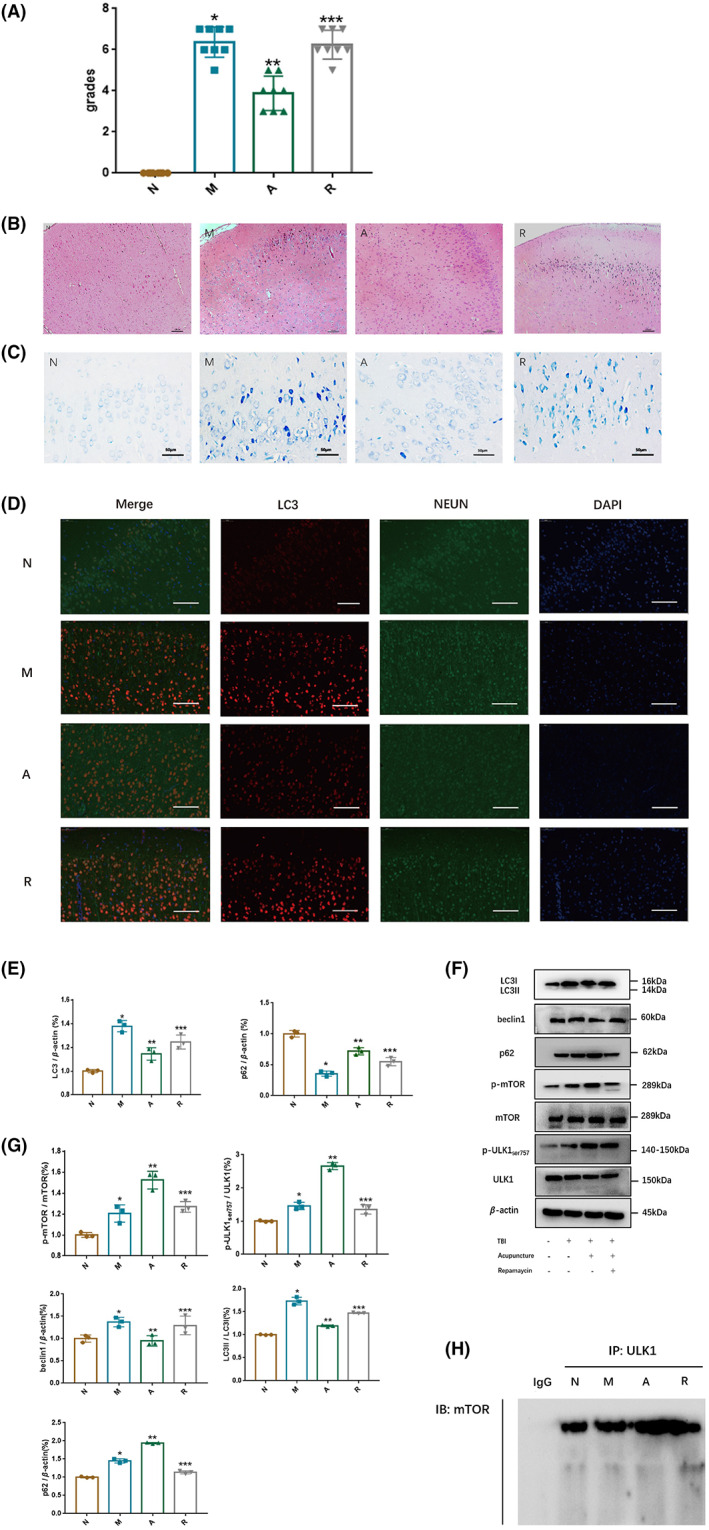
Acupuncture regulates neuronal autophagy through mTOR‐dependent signaling pathways. (A) Results of mNSS neurological function scoring; (B) HE staining (bar = 200 μm); (C) Nissl staining image (bar = 50 μm); (D) Immunofluorescence results of each group (bar = 50 μm); (E) qRT‐PCR results of LC3 and p62; (F, G) The expressions of autophagy‐related proteins were measured with Western Blot. (H) The combination of mTOR and ULK1 was determined with co‐immunoprecipitation (CO‐IP). All results were presented as mean ± standard deviation. *p < 0.05* defined group difference was statistically significant; *, **, *** defined compared with the normal group, the model group, and the acupuncture group, respectively.

To clarify the relationship between acupuncture and mTOR ‐ dependent neuronal autophagy, we treated TBI rats with acupuncture combined with rapamycin (mTOR inhibitor). Neurological grades (Figure 5A), HE staining (Figure 5B), and Nissl staining (Figure 5C) results showed that after combined treatment with rapamycin, the effect of acupuncture on alleviating neurological impairment was weakened on the 7th day after TBI and the neurons even showed nuclear pyknosis. In addition, in terms of monitoring and tracking neuronal autophagy on Day 7 of brain injury, kinds of effects in the acupuncture group were offset after treating by rapamycin combination with acupuncture (Figure 5D and Figure 5E–G). The results above suggested that the benign protective effect of acupuncture after TBI may be closely related to mTOR‐dependent signaling pathway‐mediated autophagy.

To further explore its molecular mechanism, we detected protein levels of p‐mTOR, mTOR, p‐UlK1Ser757, and ULK1 with Western blotting. As illustrated in Figure 5F and Figure 5G, acupuncture enhanced the p‐mTOR /mTOR ratio and p‐ULK1Ser757 /ULK1 ratio on Day 7 after TBI. Moreover, the CO‐IP results (Figure 5H) showed that acupuncture treatment accelerated the connection between mTOR and ULK1, while the connection was inhibited under rapamycin combination therapy. It is obvious that the activation of acupuncture on mTOR could be receded when in combination with rapamycin. Collectively, acupuncture may mediate the autophagy of cerebral cortex neurons through the mTOR/ULK1 pathway, thus exhibiting a neuroprotective effect.

## 4 DISCUSSION

Traumatic brain injury (TBI) induced functional brain impairment and neurobehavioral changes are important risk factors for multiple neurodegenerative diseases.^17^, ^18^, ^19^ Many studies have demonstrated that acupuncture plays an important role in post‐TBI neural restoration.^20^, ^21^, ^22^, ^23^ However, the biological mechanism remains to be clarified. In this work, we revealed neuronal autophagy contributes to the neuroprotective effects of acupuncture. Our study suggested that acupuncture could effectively reduce morphological damage of neurons and restore functional impairment in post‐TBI rats through benign autophagy regulation, specifically via the mTOR/ULK1‐dependent signaling pathway.

Increasing evidence demonstrated that autophagy was involved in every stage of post‐TBI neurogenesis, autophagy interacts with mechanisms such as oxidative stress, inflammation and apoptosis, which has become a neuropathological feature of post‐TBI.^24^ But it remains controversial whether it has a protective or destructive role in post‐TBI neurons. On one hand, recent studies have found that the induced autophagy pathway can reduce post‐TBI neuronal apoptosis effectively.^25^ On the other hand, some argued that inhibition of autophagy activation can effectively promote the recovery of cognitive function after TBI^.^ ^26^ In this project, we proved that the role of autophagy in TBI mainly depends on the degree of autophagy induction and the duration of autophagy activation. Surprisingly, acupuncture was able to induce or inhibit autophagy at different time points to protect neurons according to the needs of the body. Microtubule‐associated protein 1 light chain 3 (LC3) is cleaved by the protease Atg4 to form LC3‐I, then LC3‐I is conjugated to phosphatidylethanolamine (PE) to form LC3‐II, which binds tightly to the autophagosome membrane.^27^ The increase of LC3‐II not only represents the induction of autophagy but may also indicate the inhibition of autophagy degradation.^28^, ^29^ Beclin1 is an important molecule for autophagosome formation, which is then combined with VPS34, Atg14L and other various proteins to form the Beclin1 complex and controls the initiation and nucleation stages of autophagosome formation.^30^ Beclin1 and LC3 are recruited into phagosomes to perform the uptake of dead cells after autophagy activation.^31^ The interaction between p62 and LC3 accelerates cytoplasmic organoid ubiquitination into autophagosomes and is optionally degraded by autophagy.^28^ On Day 3 after TBI, neurons on the injured side exhibit autophagosome accumulation and an increased positive rate of neuronal LC3 cells. At this point, acupuncture inhibits p62 expression and activates LC3 expression. It indicates that the damaged autophagy flux may be the main cause of abnormal metabolic degradation of autophagosomes in post‐TBI. Acupuncture protects neural function after TBI by ameliorating the autophagy flux, inducing the activation of autophagy, and accelerating the cleaning of abnormal cell structures. On Day 7 and Day 14 after TBI, the cortical neurons on the injured site showed mild restoration of autophagy flux, but the structure and function of the survival neurons were destroyed by excessive autophagy. At this point, acupuncture protected the viable cells by inhibiting cell autophagy and preventing continuous cell damage or death caused by excessive autophagy.

The mammalian target of rapamycin (mTOR) is a homeostatic nutrient sensor and a central regulator of autophagy.^32^ A growing body of evidence suggests that mTOR‐dependent neuronal autophagy is a crucial target of TBI therapy.^33^, ^34^, ^35^, ^36^ Specifically, mTOR binds to several concomitant proteins and forms two signal transduction complexes, mTORC1 and mTORC2.^37^ MTORC1 is a major negative regulator of autophagy that could be bound with rapamycin to promote autophagy induction.^38^ MTORC1‐mediated phosphorylation directly blocks the complex formation between autophagy‐related gene product Atg13 and autophagy initiation protein UNC‐51‐like kinase 1 (ULK1).^32^ Also, MTORC1 could prevent AMPK‐mediated phosphorylation of ULK1 by phosphorylating ULK1 at Ser757.^10^, ^32^, ^39^ Moreover, MTORC1 could negatively regulate the transcription factor TFEB for lysosomal biogenesis and inhibits LC3 activation.^32^ The ULK1 complex occupies an important position in the initiation stage of autophagy.^12^ After autophagy induction, the ULK1 complex translocates to autophagy initiation sites and motivates the formation of the beclin1 complex, which is vital to recruiting signaling molecules. Then, multiple proteins interact to expedite the formation and expansion of phagosomes and eventually turn into autophagosomes.^12^ Our results showed that on day 3 after TBI, the pharmacological inhibitor 3‐MA attenuated the autophagy induction upon acupuncture and weakened acupuncture's inhibitory effect of mTOR/ULK1 signaling on autophagy. On Day 7 after TBI, acupuncture activated the mTOR/ULK1 signaling pathway, and the efficacy of acupuncture declined after combined treatment with rapamycin. This further demonstrated that acupuncture can regulate the initiation and nucleation stages of autophagy through the mTOR/ULK1 signaling pathway.

In the regulation of autophagy, mTOR activity is closely related to amino acids, growth factors, TSC Complex‐RHEB, AMPK, etc.^40^ It is well established that acupuncture has a multifunctional neuroprotective effect, thus the molecule mechanism of acupuncture may be multi‐factors and multi‐signal pathways.^41^, ^42^ For example, acupuncture can reduce nerve injury by miRNA‐34 /Wnt/autophagy or by Pink1/Parkin mediated mitophagy.^43^, ^44^ Therefore, we plan to conduct different experiments in future studies to verify other related pathways of autophagy, in order to perfect the regulatory mechanism of acupuncture on neuronal autophagy in TBI.

We noticed that previous studies mentioned the correlation between female age and pathological mechanisms in neurological diseases.^45^, ^46^, ^47^ However, sex differences have not been found in pediatric patients with mild TBI.^48^ Hence, the sex differences in adult patients may be due to the interaction of hormones, such as estrogen and progesterone, as well as genes.^49^ Therefore, we only used male rats in this study to rule out the effect of different hormone levels on the autophagy mechanism of TBI.

Overall, our outcomes reveal that acupuncture has time‐dependent regulation on neuronal autophagy in the damaged cerebral cortex after TBI, and both of them are neuroprotective. The neuroprotective effect may be achieved by regulating the mTOR/ULK1 signaling pathway, thus affecting the initiation of autophagy. In consequence, our results provide a new sight for the specific mechanism of acupuncture in treating TBI.

## AUTHOR CONTRIBUTIONS

Sisi Zhao, Shiqi Wang, and Luxi Cao should be considered similar in the author order of this manuscript, conducted the experiments, analyzed the data, and drafted the article. Hai Zeng and Shujun Lin performed acupuncture. Zhuowen Lin performed Western blot. Minan Chen performed the data analysis. Mingmin Zhu helped with the completion of the experiment. Yimin Zhang and Zhao Pang as the corresponding author of this manuscript designed the study and finally approved the version. All authors read and approved the final manuscript.

## FUNDING INFORMATION

This study was supported by the National Natural Science Foundation of China (grant numbers: 81873362 and 82174483); Natural Science Foundation of Guangdong Province (grant numbers: 2114050002002).

## CONFLICT OF INTEREST

The authors declare no competing financial conflict of interest.

## Data Availability

The data that support the findings of this study are available from the corresponding author upon reasonable request.
